# Supplementary material for: Are carbon emissions trading and green financial instruments synergistic? -Comprehensive quantitative research based on content analysis
Source: PLoS One. 2024 Mar 7;19(3):e0298601. doi: 10.1371/journal.pone.0298601 (PMC10919601; doi:10.1371/journal.pone.0298601)
Supplement: S1 Data — (ZIP) [file pone.0298601.s001.zip › Data/地级市Z_F工作报告.zip!/╝¬┴╓/│ñ┤║╩╨/└┤╫╘╣1⁄2╓┌║┼í╛┬φ┐╦╩2╛▌═°í┐_2009.docx]

各位代表：

　　现在，我代表市人民政府向大会报告工作，请予审议，并请列席会议的同志提出意见。

　　一、2008年工作回顾

　　2008年，我们是在克服困难和应对挑战中不断前进的。一年来，国内外经济环境发生了复杂深刻的变化，影响长春发展的不利因素明显增多。面对日益严峻的形势，在市委的正确领导下，在市人大、政协的监督和支持下，我们紧紧依靠和带领全市人民，深入落实科学发展观，坚持加快发展不动摇，继续保持了经济社会又好又快的发展态势，圆满完成了市十三届人大一次会议确定的各项任务。

　　这一年，是经济持续快速增长的一年。预计地区生产总值完成2588亿元，增长16.5%。全口径财政收入完成370亿元，增长30%。固定资产投资完成1890亿元，增长40%。社会消费品零售总额完成945亿元，增长21.4%。外贸进出口总额完成85亿美元，增长22.5%。这些主要经济指标增幅在副省级城市中均位居前列。

　　这一年，是发展能力明显增强的一年。5大类20个组团150个重大项目顺利推进，汽车、轨道客车、玉米加工、农机装备、能源产业开始了新一轮的产业扩能。南部新城、西部新城、长东北开放开发先导区启动建设，北部新城、西南工业区开发初具规模，奠定了长春未来发展的广阔空间。

　　这一年，是群众得到较多实惠的一年。城市居民人均可支配收入和农民人均纯收入分别增长13%和12.7%，城乡居民收入多年来首次同时实现两位数增长。制定实施了2008民生行动计划，办了一大批群众热切盼望的大事、实事。

　　这一年，是喜事连连、捷报频传的一年。特别是长春被正式命名为国家卫生城市、全国绿化模范城市、国家知识产权示范城市，创建“双拥模范城”实现“六连冠”，在全国大中城市畅通工程评比中位列第三名，长春的城市品牌形象和影响力都有了较大提升。

　　这些成果，标志着我市在振兴发展的进程中又迈出了坚实的一步，进一步增强了全市人民继续前进的信心和勇气。

　　一年来，我们主要做了以下七方面工作：

　　（一）提高工业运行质量，扩大工业产出能力

　　深入开展“提速增效”活动，努力保持工业健康发展势头。规模以上工业产值完成3600亿元，增长26.8%。汽车工业产值完成2353亿元，增长16.6%。农产品加工业产值完成520亿元，增长38.2%。规模以上工业企业由1003户增加到1217户。产值超亿元的工业企业由174户增加到232户。全国驰名商标数量由5件增加到10件。工业经济整体实力进一步提升。

　　全力推进汽车产业扩能，大众10万辆奥迪、轿股8万辆奔腾、轿股20万辆自主车型、丰越20万辆卡罗拉四个项目开工建设，中兴20万辆皮卡和SUV、双龙等10万辆改装车项目顺利推进。启动实施了大众发动机、一汽动力总成园等一批核心零部件项目。这些项目全部投产后，整车以及发动机等核心零部件产能均将增加一倍以上，汽车产业发展正在实现历史性的跨越。

　　加快建设一批专业化的产业园区，轨道客车整车制造、研发检测、零部件制造和车辆维修四大中心开工建设，将形成800辆铁路动车组、800辆快速城轨的生产能力。大成玉米20万吨化工醇建成投产、兴隆山园区100万吨化工醇开始设备安装。农机装备园集聚了30多户企业，长拖与世界最大的农机制造企业凯斯·纽荷兰公司实现合作。热电三厂竣工投产，九台华能、热电四厂建设进展顺利。

　　全面推进开发区发展，高新区启动超达北区基础设施建设，汽开区和经开区兴隆山区域产业集聚效应日益增强，各省级开发区、工业集中区迅速发展壮大，都形成了初具规模的项目集群。全年新开工投资3000万元以上的工业项目746个，完成工业投资800亿元，增长31.4%。这几项主要指标都创出了历史新高。

　　（二）强化“三农”工作，新农村建设迈出新步伐

　　针对春旱威胁、农资涨价等突出问题，及时出台抗旱水具补贴、化肥运销补贴等一系列措施，确保了一次播种一次拿全苗。粮食产量达到176.8亿斤，获得前所未有的大丰收。

　　启动实施新增30亿斤商品粮工程，完成72万亩的全程农机化示范区建设和312万亩的深松整地。建成了覆盖全市的人工增雨防雹作业系统，抗旱减灾能力明显增强。启动实施新增2000个健康畜禽养殖小区工程，当年新建400余个。

　　加快县域经济发展，5县（市）区生产总值增长22.2%，全口径财政收入增长30.6%，规模以上工业产值增长56.1%，工业投资增长56.3%。这些指标均明显高出全市平均水平。

　　改造泥草房2.67万户，除险加固病险水库16座，营造林5116公顷，新建农村公路2060公里，解决12.6万农村群众的饮水安全问题，农村生产生活条件得到进一步改善。

　　（三）发展现代服务业，促进服务业晋档升级

　　韩亚银行、招商银行、东北中小企业信用再担保公司相继入驻长春，邮政储蓄长春分行成立，民生银行长春分行通过银监会审批，一汽启明在深交所成功上市，金融业发展实现重大突破。

　　国内最大的汽配市场高力北方汽贸城建成开业，东北亚石陶集散中心等3个市场投入使用，新光复路市场等5个市场开工建设，区域性批发市场建设取得明显进展。

　　中信农博园、会展中心大综合馆建成投入使用，全年举办各种旅游会展活动140多项，旅游业总收入增长22%。农博会盛况空前，电影节亮点纷呈，东博会硕果累累，冰雪节、消夏节、雕塑展都办出了新特色、新水平。

　　制定出台促进房地产业平稳健康发展的意见，房地产开发施工面积增长33.5％，投资增长55.5%。新建150万平方米标准化厂房，工业地产开发渐成规模，土地节约集约利用水平进一步提高。

　　（四）推进改革创新，扩大对外开放

　　国企改革收尾工作进展顺利，7户企业完成破产终结，25户企业破产清算取得实质性进展。长拖、电炉、柴油机等改制企业与战略投资者实现合资合作，一批老字号企业重新焕发活力。民营经济快速发展，已经成为拉动全市经济增长的重要力量。

　　启动汽车及关键零部件开发与制造等8个重大科技专项，稀土镁合金等67项关键技术研发取得突破性进展。光电子和生物两个国家级产业基地建设顺利推进。科技成果转化和高新技术产业化进程不断加快，高新技术产值增长25％。

　　深入开展招商引资“九个月攻坚”活动，引进爱尔兰CRH等5户世界500强企业，在长投资的世界500强企业达到51户。与13户中央企业合作取得实质性进展，37户外资企业增资扩股，全年实际利用内外资均增长20%以上。

　　努力扩大对外出口，出口总额实现17亿美元，增长12.6%。机电产品和高新技术产品分别增长33.4%和32.3%，出口结构进一步优化。内陆港建成投入使用，长春拥有了方便快捷的出海通道。

　　（五）加强基础设施建设，扎实创建国家卫生城

　　编制完成了中心城区380平方公里的控制性详细规划。土地管理和利用工作得到加强。全年新建续建14座桥梁、117条道路，精细化改造278条街路，轻轨三期开工建设，长吉城际铁路、哈大客运专线顺利推进，长双烟铁路竣工通车。

　　新植树木20万株，绿化街路30条，新建改造10个公园、165块大块绿地。石头口门水源地保护工程顺利启动，栽植芦苇145公顷。南部污水处理厂通水试运行，东南污水处理厂和4县（市）污水处理厂开工建设。集中整治烟囱、锅炉房608座，空气质量优良级天数继续保持340天以上。

　　城区居民楼道、主要公园亮化基本实现全覆盖，火车站等6个重点区域和人民大街等44条街路实现高标准亮化。紧急抢建输气管线和脱碳装置，新增60万立方米的日供气能力。新建改造供水管线69.1公里，新增集中供热面积350万平方米。

　　深入开展城市清洁行动，全面提高环卫作业标准，不断强化重点商圈、主要街路的日常保洁。粉刷街路两侧楼体9.2万平方米，清刷覆盖非法广告12万余处，一些城市管理顽疾基本消除，精细化管理的长效机制初步建立。

　　（六）全面实施民生行动计划，努力改善民生状况

　　新增城镇就业8.6万人，扶持就业困难群体就业1.6万人, “零就业家庭”保持动态为零。较好地解决了灵活就业人员、农民工参保续保问题，养老保险新增8.6万人，失业保险新增8万人，全年发放养老金、失业保险金38.4亿元，保障了40万名离退休职工和失业人员的基本生活。新征地农民养老保险实现应保尽保。

　　较大幅度提高重点优抚对象补助标准、重症贫困精神病人救助标准、城区农村低保对象补助标准，向城乡低保群众发放临时生活补贴，在物价大幅波动的情况下确保了困难群众生活水平不降低。

　　加强公共交通设施建设，新建166座公交候车亭，更新公交车387辆，为7万多名65岁以上的残疾人和70岁以上老人办理免费乘车IC卡。整顿规范了出租车行业管理秩序，进一步降低了出租车从业人员的负担。

　　进一步扩大医保覆盖面，城镇居民医保新增25万人，参保率达到93%；新农合新增17.6万人，常住人口参合率达到98.9%。城乡医保覆盖面分别高出国家基本全覆盖标准8个和13.9个百分点。117个社区卫生服务机构、145个乡镇卫生院基本完成标准化建设。全面加强困难群众医疗救助力度，407名唇腭裂患儿、3600多名贫困白内障患者得到免费救治。

　　建设7所农村寄宿制学校，改善5所非改制学校办学条件。启动 “大学区”模式改革试点，70多所优质校与薄弱校开展全方位对口帮扶。培训2000名农村骨干教师，建设100所农村中小学远程教育示范校。加强5县（市）区职教中心建设，全市完成职业培训54.1万人次。

　　启动艺术剧场、话剧院、杂技宫修缮工程，科技文化中心建设进展顺利，烈士纪念馆、图书批发大厦、图书馆铁北分馆、宽城区文化馆落成投入使用。农家书屋初级建设实现了全覆盖，335个自然村实现了广播电视“村村通”。搭建9个露天舞台，组织广场活动200场，放映数字电影20000余场，深入开展欢乐庄稼院、社区艺术节等活动，群众文化生活日益活跃。

　　低保家庭住房租赁补贴范围由8平方米提高到11平方米，享受补贴的家庭由7127户增加到8358户，户均补贴标准提高30%。购买新建7.3万平方米廉租房，新建5万平方米廉价房、23.5万平方米经济适用房，解决了6230户低收入家庭的住房困难。改造246万平方米棚户区及危旧房屋，4万余户居民改善了住房条件。

　　在供暖成本大幅提高的情况下，适度提高了供暖费标准。为保障困难群众的冬季供暖，继续实行低保户集中供热费全额减免政策，自行取暖的低保户采暖补贴提高了60%，低保边缘户开始享受采暖补贴。紧急接收125处弃管锅炉房，确保24000多户居民正常供热。

　　采取政府贷款贴息、承债、垫资等多种方式，解决困扰我们多年的历史遗留问题，600多名改制企业困难职工接续社保，38000多名原国企退休人员办理医保，2万余户临时用电家庭实现安全用电，3万余户交费未开栓燃气用户开栓供气，4.1万户无籍房开始办理产权证，11万户居民用上了放心水。

　　（七）加强政府自身建设，保持社会和谐稳定

　　认真执行市人大及其常委会的决议、决定，自觉接受市人大及其常委会的监督，主动接受市政协的民主监督，办理人大议案、建议和政协提案517件。深入开展“五·五”普法，依法治市工作不断加强。

　　全面开展了解放思想大讨论活动，加快推进政府工作创新，行政审批项目和年审年检项目减放比例达到63％和77%，保留的审批权限基本都集中到政务大厅，审批程序明显简化，审批效率进一步提高。

　　深入开展以精细化管理为主题的“查找改”活动，政府各部门对管理粗放问题进行全面排查，对查出的795个问题逐项落实整改措施，并建立巩固成果的长效机制，政府管理水平得到进一步提高。

　　认真落实领导干部读报制度，全年共解决媒体反映的各种问题1143个。通过读报掌握社情民意、弥补工作缺失、满足群众诉求，已经成为政府各部门的自觉行动。全面加大信访工作力度，妥善处理了一批涉及群众利益的难点问题。

　　加强城市应急能力建设，有效控制了风疹、手足口病疫情，妥善处置了问题奶及其制品事件。110、119、122实现三台合一。除极特殊情况外，110、119出处警和120救护车到达现场时间分别缩短到5分钟和12分钟以内。火灾防控和灭火救援能力明显增强，成功扑救了汉斯啤酒城等火灾，有效防止了重大火灾事故的发生。

　　深入开展社会治安综合治理，刑事案件破案率提高6个百分点，命案破案率达到92.9%，交通肇事逃逸案破案率达到90%，实现了奥运圣火传递和奥运期间安保任务万无一失的目标。公安机关“三基”工程建设被公安部评为优胜单位，连续三届被授予“全国社会治安综合治理优秀地市”称号。

　　四川汶川特大地震发生以后，全市上下情系灾区、踊跃捐助，累计捐赠款物折合人民币1.2亿多元，先后派出7支队伍600多名救援人员赶赴第一线开展抢险救灾和对口援建。长春人民用爱心和真情，为灾区同胞送去了抗震救灾、重建家园的勇气和力量。

　　过去一年，审计、外事、侨务、气象、防震、方志、档案、民兵、预备役等工作得到加强，民族宗教、新闻出版、人口计生、妇女儿童等工作也取得长足进步。

　　各位代表，在各种不利因素明显增多的情况下，过去一年能够取得这些成绩，确实来之不易。这是市委正确领导的结果，是人大、政协监督支持的结果，是长春750万人民共同奋斗的结果。在此，我代表市人民政府，向为长春发展做出无私奉献、付出辛勤劳动的全市人民致以亲切问候和崇高敬意！向给予政府工作大力支持的人大代表和政协委员，向各民主党派、工商联、无党派人士和社会团体，向人民解放军驻长部队和武警官兵，向中央和省驻长单位，向所有关心、支持长春发展的海内外朋友们，表示诚挚的感谢！

　　总结一年来的工作，我们也清醒地认识到，经济社会发展和政府工作中还存在许多问题：

　　——保持经济持续快速增长的压力较大。受国际国内经济发展环境变化影响，从7月份开始我市部分经济指标增长速度逐步回落，企业市场需求下降，项目建设进度受到影响。

　　——长期形成的结构性矛盾表现仍然比较突出。经济转型和产业结构调整的任务还很艰巨，经济增长的资源环境代价还很大，人才资源尚待充分挖掘，科技成果转化能力有待进一步提高。

　　——涉及群众切身利益的问题需要进一步解决。征地拆迁方面遗留问题较多，城镇居民收入水平相对偏低，农民持续增收较为困难，贫困群众的生活状况亟须改善，服务残疾人的能力亟待增强。

　　——城市安全水平有待提高。公共安全事故、灾害事故隐患较多，城市基础设施承载能力尚需进一步提高，突发事件应急处理能力有待加强，全社会安全防范和自救能力较弱。

　　——政府自身建设需要加强。工作创新和职能转变还不够，发展软环境有待进一步优化。个别工作人员公仆意识、责任意识和服务意识不强。预防和惩治腐败工作还需要进一步加强。

　　对于这些矛盾和问题，我们将采取更加有力的措施，认真加以解决。

　　二、2009年工作安排

　　2009年，将是充满机遇而又富于挑战的一年。中央实施积极的财政政策和适度宽松的货币政策，出台了保增长、扩内需、调结构的一系列措施。长春的工业化、城市化进程不断加快，产出能力正在迅速提高，“三城两区”的发展格局全面拉开，多年积蓄的发展能量将逐步释放。这些为明年继续加快发展创造了有利条件。同时也要清醒地看到，国际经济出现了整体下滑趋势，国内经济增长的不确定因素可能继续增加。长春作为一个产业结构以汽车为主的城市，对外部形势的变化较为敏感，经济增长的压力前所未有，我们可能要遭遇改革开放以来最为严峻的考验。如何在比较困难的情况下，充分利用各种有利条件，把严峻考验转化为发展机遇，是我们面临的巨大挑战。我们必须以更坚定的决心、更昂扬的斗志、更有力的措施，扎实做好工作，奋力渡过难关，把长春的振兴发展不断推向前进。

　　明年政府工作的基本思路是：以邓小平理论和“三个代表”重要思想为指导，全面贯彻党的十七届三中全会、省委九届六次全会、市委十一届四次全会精神，深入落实科学发展观，坚持工业化、城市化“双轮驱动”的发展战略，牢牢把握加快发展、改善民生、建好城市、促进和谐的总体要求，积极争取中央保增长、扩内需、调结构的各项政策支持，更加注重促进增长稳定经济大局，更加注重着眼长远积累发展能力，更加注重统筹协调实现全面发展，更加注重改善民生维护群众利益，更加注重化解风险促进社会和谐,努力推动经济社会又好又快发展。

　　明年经济和社会发展主要预期目标是：地区生产总值增长15%左右，全口径财政收入同步增长，固定资产投资增长30%左右，万元GDP能耗降低5%左右。

　　重点抓好以下十项工作：

　　（一）集中力量做大做强工业

　　制定出台支持工业企业的相关政策，动员全市上下聚焦工业、服务工业、发展工业，形成调动各种要素促进工业增长的发展局面。举全市之力支持一汽加快发展，扶持大成、长客等重点企业做大做强。加大帮扶解困力度，千方百计地帮助中小企业保生产、保市场、保增长。支持企业实施技术创新、强化内部管理、提升整体素质，在应对危机中转型升级，在市场竞争中发展壮大。抓好生产要素组织调度，增加流动资金供给，为工业企业生产经营创造良好条件。规模以上工业产值增长17％左右。

　　全力实施5大类20个组团150个重大项目。继续推进整车扩能，尽快实现新增100万辆整车、100万台发动机、100万台变速器、10万辆改装车、1000亿元零部件产值的目标，建设名副其实的国际汽车城。突出抓好大成玉米300万吨化工醇等一批农产品加工项目，全力打造千亿元产值的第二大支柱产业。加快建设轨道客车、农机装备、中冶京诚等产业园区和电力扩能工程，努力培育具有长期竞争优势的先进生产能力。继续开展招商引资“九个月攻坚”活动，努力引进和生成重大工业项目。全年实施投资超千万元工业项目力争达到1000个，工业投资完成1000亿元。

　　充分发挥开发区工业化的主力军作用，支持高新、经开、汽车开发区立足自身优势，多上快上工业大项目。各省级开发区、工业集中区都要按照新招50个、在建50个、竣工50个项目的模式，加快开发建设进度，尽快形成产业规模。采取更加有力的措施，吸引汽车产业、高新技术产业向西南集聚，推动西南工业区向更大规模、更高层次迈进。坚持基础设施先行，以工业项目建设为重点，建设长东北开放开发先导区，使之成为长春工业未来的重要支撑。

　　（二）推进新农村建设

　　加大对农业农村支持力度，建立健全以城带乡、以工补农的长效机制。调整财政支出结构，对农业投入增长幅度要高于经常性收入增长幅度。大幅度增加农村基础设施、社会事业、公益性项目的财政投入，大幅度提高政府土地出让收益、耕地占用税新增收入用于农业的比例。

　　增强县域综合生产能力。实施增产30亿斤商品粮工程，抓好3大灌区改造、450万亩耕地深松整地、450万亩全程机械化示范区建设。推进种植业结构调整，集中连片建设特色经济作物园区。实施2000个健康畜禽养殖小区建设工程，当年新建400个左右。加快小城镇建设，每个县（市）区都要建设2到3个经济强镇。加快推进农村工业化，县域工业投资增长30%以上，经济总量和财政收入均增长20%以上。

　　创新农业农村发展模式，稳步推动土地向规模大户集中、牧业向养殖小区集中、工业项目向园区集中、农民向城镇集中。推进涉农事业单位改革，建立新型农业技术推广体系。鼓励和引导农民发展新型农村合作经济组织，大力发展农业产业化经营。保障农民合法权益，推进农村民主法制建设。

　　努力改善农村生产生活条件，改造泥草房4万户，解决25万农村群众饮水困难，新建210个农村社区工作站，基本完成31座病险水库除险加固，完成营造林5000公顷。实施 “新网”工程，建立农村现代流通服务体系。基本完成 “村村通”公路建设，逐步扩大“屯屯通”覆盖率。

　　（三）发展壮大服务业

　　改造提升传统服务业。改造重庆路步行街，建设红旗街文化商贸一条街，发展一批特色商业街。加快建设20个区域性批发市场，新光复路市场等4个市场建成营业，汽车拆解市场等4个市场开工建设。启动建设10个商务综合体，建设一批五星级酒店，提高城市承接能力。

　　大力发展现代服务业。加快建设铁北物流园区，启动陆路干港二期建设。继续引进国内外金融机构入驻长春，规划建设南部新城金融区。加快引进和培育服务外包企业，建设服务外包产业园区，争取成为国家级服务外包基地城市。办好东博会、汽博会、农博会、民博会、冰雪节、消夏节、中日韩友城大会等重点展会。加强旅游资源开发，推进最佳旅游目的地城市建设。

　　稳定发展房地产业。全面落实财政支持、简化手续等22项优惠政策，鼓励支持人民群众改善住房条件。加快无籍房屋确权进度，扩大住房交易总量，激活二手房市场。积极引进国内外有实力的开发商，发展商业地产和工业地产，保持住宅地产的稳定健康发展势头，力争房地产开发面积、销售面积不低于今年水平。

　　（四）建设绿色宜居城市

　　加强规划控制和引导，抓好新城开发和老城区改造。编制完成城市规划区控制性详细规划、全市土地总体利用规划。南部新城，建设核心区基础设施，启动一批重大项目。北部新城，实现凯旋路全线贯通，启动周边区域开发。西部新城，开工建设西客站及周边基础设施。老城区，理性科学地实施改造，全面提升城市品位和形象。严格保护耕地，强化节约集约用地。

　　加强道路基础设施建设，缓解交通紧张状况。续建四环路，实施亚泰大街、台北大街快速路改造，全面推进主次街路渠化改造，综合治理重要交通节点，逐步推广单行线和右转直行，加强静态交通管理。落实公交优先发展政策，提高公共交通分担率。建设轻轨三期，抓紧地铁一号线前期工作并争取尽快启动。续建长吉城际铁路、哈大客运专线，启动龙嘉机场扩建工程。加快构建通达省内市（州）的高速公路网。

　　加大园林绿化力度，促进人与自然相和谐。新城区要同步推进绿化建设，老城区要见缝插绿，千方百计增加绿量，进一步塑造森林城的形象。新建一批绿化精品，增加花卉种植面积，逐步形成一街一景的景观效果。继续改造动植物园、长春公园。友谊公园、裕华园建成对游人开放。加快推进净月潭退耕还林和水域治理，继续整治伊通河生态河道，完成机场路风景林、长双公路绿化带建设。

　　深入推行精细化管理，巩固创建卫生城成果。综合整治户外广告，打击非法广告和清理不良广告，确保广告内容格调健康。加大依法管理力度，建立市容管理长效机制。出台新的物业管理办法，提高物业管理水平。合理划分市区两级权限，向基层放权力、给条件、落责任，进一步发挥城区、开发区在城市管理中的作用。

　　（五）加快科教兴市步伐

　　加强科技创新，建设创新型城市。积极推进清洁能源汽车等重大科技项目转化，着力突破制约产业转型升级的关键技术。充分调动在长院校、科研院所的积极性，发挥企业在自主创新中的主体作用，加强产学研合作，促进经济与科技有效结合。发展大学科技园、中外科技合作产业园区等各类孵化器，壮大光电子和生物两个国家级产业基地，加快高新技术产业化进程，高新技术产值增长20%以上。

　　促进教育均衡，办人民群众满意的教育。农村，中学要加快建设寄宿制学校，小学要加强中心校建设。城区，要实施中小学校标准化建设工程，尽快让每一所义务制教育学校都能拥有符合标准的硬件设施、办学水平和师资队伍。加大特殊教育支持力度，康复设施年内全部达标。深化中小学办学体制改革，加强教师队伍建设，提高素质教育水平。

　　开发人力资源，提高劳动者素质。营造良好的人才成长环境，加大高层次、高技能人才引进和培养力度，努力开创人尽其才、人才辈出的发展局面。做大做强职业技术学院，改扩建2到3所中等职业学校，完善5县（市）区职教中心。异地新建一汽高等专科学校。培训下岗失业人员2万人，培训后就业率达到60%以上。对10万名农民实施技能培训，推动劳务输出由体力型向技能型转变。

　　（六）繁荣城市文化

　　全面树立20世纪遗产保护意识，恢复长影老厂区历史风貌，保护性修缮二道沟邮局中共地下党活动旧址、沙俄铁路附属地旧址等一批历史建筑和文化街区，将一汽老厂区、柴油机厂生活区纳入保护利用体系，塑造一批延续历史文脉的特色城市空间。把文化融入城市建设之中，让规划更具特色、建筑多出精品，努力使今天的杰作成为明天的文化遗产。

　　加快建设文化基础设施，艺术剧场、话剧院、杂技宫、群众文化活动中心、科技文化中心建成投入使用。鼓励和支持社会各界投资兴建一批体现城市文化和地方特色的博物馆、展览馆，丰厚城市文化底蕴。加快建设乡镇文化站、村文化室，努力提高农家书屋建设水平。

　　努力发展文化产业。加大对文化企业的扶持力度，培育一批知名文化品牌。积极推进净月科技文化服务区、吉林动漫游戏原创产业园、双阳文化印刷产业开发区建设，启动建设朝阳创意产业示范区、光复路文化产业园、工业设计产业园。深入开展“扫黄打非”，加强文化市场管理。

　　大力发展群众文化，为基层送戏100场，举办广场文化活动200场，放映数字电影20000场。深入开展欢乐庄稼院活动，举办文化艺术周、社区艺术节和农民文化节。深化文化体制改革，大力发展文化公益事业，保障人民群众的基本文化权益。充分发挥群众文化的教化陶冶功能，弘扬“宽容大气、自强不息”的城市精神。

　　（七）全力保障和改善民生

　　把“创新、深化、务实”作为开展民生工作的总要求，更加重视普通群众意愿，更加突出中低收入群体，更加关注经济调整时期群众的生活困难，集中力量为人民群众办一批实事。

　　千方百计增加居民收入特别是农民收入，进一步提高企业退休人员基本养老金水平和城乡低保群众、优抚对象的生活补助标准。实施更加积极的就业政策，全方位促进就业增长，确保就业形势基本稳定。创新就业岗位开发模式，建立就业再就业工作的长效机制，新增城镇就业8.8万人。保持“零就业家庭”动态为零。

　　推进社会保险扩面攻坚，养老、失业保险分别新增参保人数5万人。继续采取政府贴息贷款和低息贷款两种方式，帮助特殊困难人员接续基本养老保险。开展农村独女户养老保险试点，新失地农民养老保险实现应保尽保。统筹社会资源建立城乡大救助体系，全方位、多层次地改善困难群众基本生活条件，及时将陷入困境的群众纳入社会救助体系，确保每一位群众都有必需的生活来源和基本的生活保障。

　　加强残疾人职业技能培训，有针对性地开发残疾人就业岗位，新增城镇残疾人就业2000人。提高重度残疾人的低保补助标准。优先保障残疾人住房需求。加强无障碍设施建设。全面加大聋儿、盲人、肢残群众、重症精神病人的康复救助力度，对贫困家庭的白内障患者、先天性心脏病儿童患者、唇腭裂儿童患者康复手术实施“有一助一”。努力营造人人献爱心、人人关爱残疾人的社会环境。

　　把住房保障作为政府工作重要任务，购买和新建12.5万平方米廉租房，新建30万平方米廉价房，改造200万平方米棚户区及危旧房，力争用三年时间基本解决当期低收入群众的住房困难。突出抓好回迁房建设，确保当期回迁率达到100%。逐步推广先安置、后拆迁的征地拆迁模式，绝不允许违法暴力拆迁，依法保护被拆迁居民利益。

　　（八）提高城市健康水平

　　全面实施“健康长春”行动计划，深入开展健康促进工作，持续不断地提高城市健康水平，努力让人民群众享受健康的快乐。

　　培育健康人群。广泛开展健康知识教育宣传活动，积极控制各种致病因素，加强重点疾病早期筛查，倡导文明健康的生活方式。开展1000项以上全民体育健身活动，建设2个体育健身园，为13个乡镇、164个行政村安装健身器材，全面提高群众身体素质。

　　塑造健康家庭。实施优生优育促进工程，建立全过程的新生儿出生缺陷干预和先天性疾病治疗体系。新生儿出生缺陷发生率五年内降低到4‰以下。加强社区卫生保健服务，城市常住人口健康档案建档率达到80%以上。深入实施“助老工程”，提高老年人的健康水平。

　　营造健康环境。积极推进节能减排，削减化学需氧量和二氧化硫排放，大力推广清洁生产。建设热电集中供热工程、蘑菇沟垃圾处理场，加快建设东南污水处理厂和4县（市）污水处理厂。实施石头口门水源地植苇净水工程，栽植芦苇10平方公里。规划建设西北部生态经济区和一批城市生态湿地公园。

　　优化健康服务。全面提高乡镇卫生院、社区卫生医疗机构的装备水平和诊疗能力，50%以上的村有标准化卫生所(室)，为人民群众提供安全、有效、方便、价廉的医疗卫生服务。继续扩大城镇居民医疗保险和农村新农合覆盖面，稳步提高补贴标准和报销比例。建立城镇居民医疗保险与城镇职工医疗保险相衔接的制度。

　　（九）加强城市安全建设

　　贯彻安全第一、预防为主、综合治理的方针，在全市范围内深入持久地开展安全年创建活动，直至公众安全意识全面提高、安全长效机制基本形成、社会安全形势根本好转。

　　食品安全，以肉蛋奶、米面油、瓜果蔬为重点，建立从农田到餐桌的全过程的认证、检测和监管体系，全面整顿食品行业市场秩序，确保人民群众吃上合格、放心的食品。

　　生产安全，加大投入、强化监管，达不到安全生产条件的企业要坚决进行整改，绝不允许以损害人民健康、甚至牺牲职工生命谋取利益。安全生产事故发生率、死亡人数保持零增长，并力争有所下降。

　　消防安全，继续加强公共消防设施和消防设备建设，加大火灾隐患的排查治理力度，从源头遏制重特大灾害事故的发生，重大火灾隐患整改销案率力争达到100%。

　　交通安全，加快推进交通管理设施改造，严把驾驶员培训考试关，从严整治机动车行车秩序，努力增强全社会遵守交通规则的自觉性。严打交通肇事逃逸行为，采取更加有力的措施巩固和提高交通肇事逃逸案件侦破率。

　　治安安全，认真落实社会治安打防控措施，突出抓好科技强警，构建城区治安防控网络，加大刑事治安案件的侦破力度，进一步提高人民群众的安全感。长春绝不允许任何一种治安问题成风，绝不允许黑恶势力滋生做大。

　　基础设施安全，加强水、电、气、热设施改造，抓好防洪、抗震设施建设，建立保障有力的应急物资储备和应急救援队伍，全面提高城市运行的可靠性和防灾减灾能力。做好四川汶川地震对口援建工作。

　　（十）推进政府自身建设

　　坚持依法行政，建设法治政府。自觉接受市人大及其常委会的监督，主动接受市政协的民主监督，定期向市人大及其常委会报告工作，向市政协通报情况、征询意见。深入开展双拥共建活动，增进军政军民团结。规范行政执法行为，不断提高依法行政能力，优化经济发展软环境。

　　加快改革创新，建设服务政府。深入开展学习实践科学发展观活动，认真搞好“查找改”，着力破解体制、机制和工作作风方面存在的突出问题。实施政府机构改革，理顺部门职责关系，强化社会管理和公共服务职能。深入开展读报活动，办好市长公开电话，为群众解难题、办实事、谋福祉。

　　转变工作作风，建设务实政府。创新工作方式，把“项目化”作为推进工作的重要载体，所有工作都要有目标、有标准、有监督、有检查。实施行政首长问责制，全面加大督查工作力度，形成雷厉风行、令行禁止的风气，确保政府的各项工作落到实处。

　　坚持从严治政，建设廉洁政府。严格执行廉洁从政各项规定，厉行节约，压缩开支，降低行政成本。加快推进教育、制度、监督并重的惩治和预防腐败体系建设，确保权力在阳光下运行。严肃查处各类违法违纪案件，坚决纠正损害群众利益的不正之风。

　　明年是本届政府工作的非常时期，非常时期要有非常作为。所有政府组成人员都要朝气蓬勃地做好工作，奋发有为地成就事业，做克己奉公、勤奋敬业的表率；都要深入基层为企业排忧解难，与企业一起共克时艰，做恪尽职守、服务大局的表率；都要倍增爱民之心，竭尽为民之责，做艰苦奋斗、勤政为民的表率。

　　各位代表！在新的形势下，把长春振兴发展大业持续推向前进，是摆在我们面前光荣而艰巨的历史任务。让我们高举中国特色社会主义伟大旗帜，更加紧密地团结在以胡锦涛同志为总书记的党中央周围，在中共长春市委的领导下，万众一心，开拓奋进，为建设繁荣、和谐、开放、美丽的新长春而不懈奋斗！
